# Initial results of a novel technique of clipped node localization in breast cancer patients postneoadjuvant chemotherapy: Skin Mark clipped Axillary nodes Removal Technique (SMART trial)

**DOI:** 10.1002/cam4.2848

**Published:** 2020-01-22

**Authors:** Geok Hoon Lim, Sze Yiun Teo, Mihir Gudi, Ruey Pyng Ng, Jinnie Pang, Yia Swam Tan, Yien Sien Lee, John C. Allen, Lester Chee Hao Leong

**Affiliations:** ^1^ Breast Department KK Women's and Children's Hospital Singapore Singapore; ^2^ Duke‐NUS Medical School Singapore Singapore; ^3^ Department of Diagnostic & Interventional Imaging KK Women's and Children's Hospital Singapore Singapore; ^4^ Department of Pathology and Laboratory Medicine KK Women's and Children's Hospital Singapore Singapore; ^5^ Division of Nursing KK Women's and Children's Hospital Singapore Singapore; ^6^ Duke‐NUS Medical School Centre for Quantitative Medicine Singapore Singapore; ^7^ Department of Diagnostic Radiology Singapore General Hospital Singapore Singapore

**Keywords:** axillary lymph node dissection, axillary staging, breast cancer, neoadjuvant chemotherapy, targeted axillary dissection, ultrasound visible clip

## Abstract

**Purpose:**

Removal of clipped nodes can improve sentinel node biopsy accuracy in breast cancer patients post neoadjuvant chemotherapy (NACT). However, the current methods of clipped node localization have limitations. We evaluated the feasibility of a novel clipped node localization and removal technique by preoperative skin marking of clipped nodes and removal by the Skin Mark clipped Axillary nodes Removal Technique (SMART), with the secondary aim of assessing the ultrasound visibility of the various clips in the axillary nodes after NACT.

**Methods:**

Invasive breast cancer patients with histologically metastatic axillary nodes, going for NACT, and ≤3 sonographically abnormal axillary nodes were recruited. All abnormal nodes had clips inserted. Patients with M1 disease were excluded. Post‐NACT, patients underwent SMART and axillary lymph node dissection. Specimen radiography and pathological analyses were performed to confirm the clipped node presence. Success, complication rates of SMART, and ultrasound visibility of the various clips were assessed.

**Results:**

Twenty‐five clipped nodes in 14 patients underwent SMART without complications. The UltraCor Twirl, hydroMARK, UltraClip Dual Trigger, and UltraClip were removed in 13/13 (100%), 7/9 (77.8%), 1/2 (50.0%), and 0/1 (0%), respectively (*P* = .0103) with UltraCor Twirl having the best ultrasound visibility and removal rate. Removal of three clipped nodes in the same patient (*P* = .0010) and deeply seated clipped nodes (*P* = .0167) were associated with SMART failure.

**Conclusion:**

Skin Mark clipped Axillary nodes Removal Technique is feasible for removing clipped nodes post‐NACT, with 100% observed success rate, using the UltraCor Twirl marker in patients with <3 not deeply seated clipped nodes. Larger studies are needed for validation.

## Graphical abstract

Skin Mark clipped Axillary nodes Removal Technique (SMART) is feasible for removing clipped nodes post neoadjuvant chemotherapy, with 100% observed success rate, using the UltraCor Twirl marker in patients with <3 not deeply seated clipped nodes.

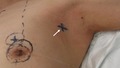

## 1 INTRODUCTION

In breast cancer patients with nodal disease, neoadjuvant chemotherapy (NACT) could result in pathological complete resolution of these metastatic lymph nodes in about 40% of patients, with a higher reported rate of 60%‐70% in human epidermal growth factor receptor 2 (HER2) positive patients.^1^ This high rate of nodal downstaging has led to the consideration of sentinel lymph node biopsy (SLNB) to stage the axilla post‐NACT, hence avoiding an axillary lymph node dissection (ALND) and its complications, which include arm lymphedema,^2^ in patients who had responded well to chemotherapy.

However, performing SLNB alone in these patients post‐NACT has a false negative rate of 12.6%‐14.2%.^3^, ^4^ Also, it was found that in about 23% of cases, the sentinel lymph nodes did not correspond to the initially metastatic clipped node.^5^ As a result, to reduce the false negative rate,^1^, ^5^ the metastatic nodes could be clipped and removed at the same setting as SLNB during a targeted axillary dissection.

Various methods of localizing a clipped node exist but all have limitations and disadvantages. The MARI procedure (marking the axillary lymph node with radioactive iodine [I] seeds), which uses a radioactive I seed as a localization agent, results in radiation exposure.^6^ Wire localization may be associated with wire migration/breakage and patient discomfort^7^ and is not always technically possible. Savi Scout reflector‐guided localization^8^ and magnetic seed localization (Magseed)^9^ are promising devices but are expensive and not readily available worldwide. To date, several ongoing worldwide trials are still assessing the feasibility of radioactive seed (Radioactive Iodine Seed localization in the Axilla with the Sentinel node – RISAS trial^10^), Magseed (ClinicalTrials.gov identifier: NCT03718455) and scout reflector (ClinicalTrials.gov identifier: NCT03281720), etc as localizing agents for the clipped node post‐NACT.

To overcome the disadvantages associated with current modalities, we introduce a novel technique of localizing and removal of the clipped node post‐NACT called Skin Mark clipped Axillary nodes Removal Technique (SMART). In this study, we aimed to evaluate the feasibility and safety of SMART with the use of different clips by assessing identification and removal rates of clipped nodes. The SMART complication rate was evaluated. Our secondary aim was to assess the ultrasound visibility of the different clips in the axillary lymph nodes, post‐NACT.

## 2 MATERIALS AND METHODS

In this prospective study, invasive breast cancer patients (T0‐4) with histologically proven metastatic axillary lymph nodes and up to three abnormal lymph nodes seen on ultrasound, suitable for NACT were enrolled. Patients with stage IV disease, four or more abnormal lymph nodes seen on ultrasound, unfit for or declined chemotherapy were excluded.

A lymph node was defined as abnormal if it had any of the following sonographic features: cortical thickness more than 3 mm, eccentric cortical thickening of more than 2 mm, or marked fatty hilar effacement. Histology of all the abnormal looking lymph nodes was obtained by core needle biopsy or fine needle aspiration cytology prior to the start of NACT. These nodes were then each clipped with a clip in a second procedure after the diagnosis of pN+ was established. In patients with multiple abnormal lymph nodes, different clips, namely UltraCor Twirl, HydroMARK, UltraClip Dual Trigger or UltraClip markers, were inserted into the cortex of each node to aid individual node identification. The choice of clip was dependent on individual radiologist preference, and six breast radiologists with 2‐15 years of experience were involved in the insertion and skin marking of the clips.

Patient demographics and measurement of the perpendicular distance between the skin and the clips post‐chemotherapy were recorded. The largest dimension of the lymph node and cortex sizes pre‐ and post‐NACT were measured as well.

The patients then underwent anthracycline and taxane‐based NACT. All HER2‐positive patients received immunotherapy. Post‐NACT, the patient had her breast surgery as planned, with removal of the clipped nodes using the novel SMART technique and ALND.

The identification rate of the different clips, successful removal rate of the clipped nodes using SMART and complication rate of SMART were assessed.

### 2.1 Surgical technique (SMART)

**Figure 1:**
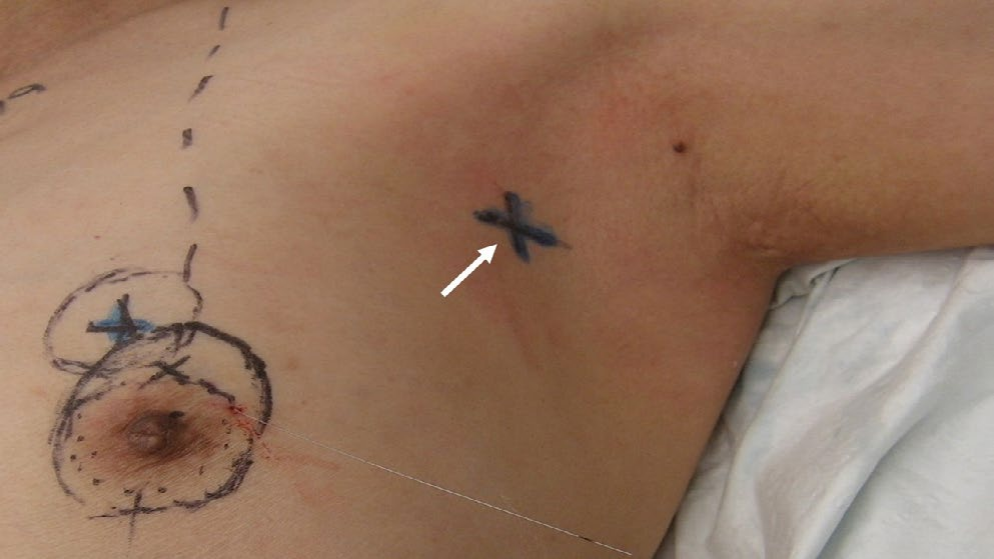
The location of the clip was preoperatively skin‐marked with a cross (arrow) with the patient's arm abducted in 90°. The patient also had a hookwire localization of her breast cancer and the markings on her breast were for a round block procedure

The position of the clip was marked on the skin preoperatively by the radiologists on the day of surgery with the ipsilateral arm abducted at 90° (Figure 1), mimicking the position of the arm during the axillary operation. To perform the skin marking, the ultrasound probe was placed perpendicularly to the skin, using one of two systems: (a) Philips IU 22 (Philips Medical Systems) or (b) Siemens S2000 (Siemens Medical Solutions) with a linear transducer of a bandwidth of 5‐12 mHz and 5.5‐18 mHz, respectively. The clip was identified based on clip morphology, its perpendicular distance from the skin, and its position relative to surrounding structures based on the pre‐NACT diagnostic ultrasound.

**Figure 2:**
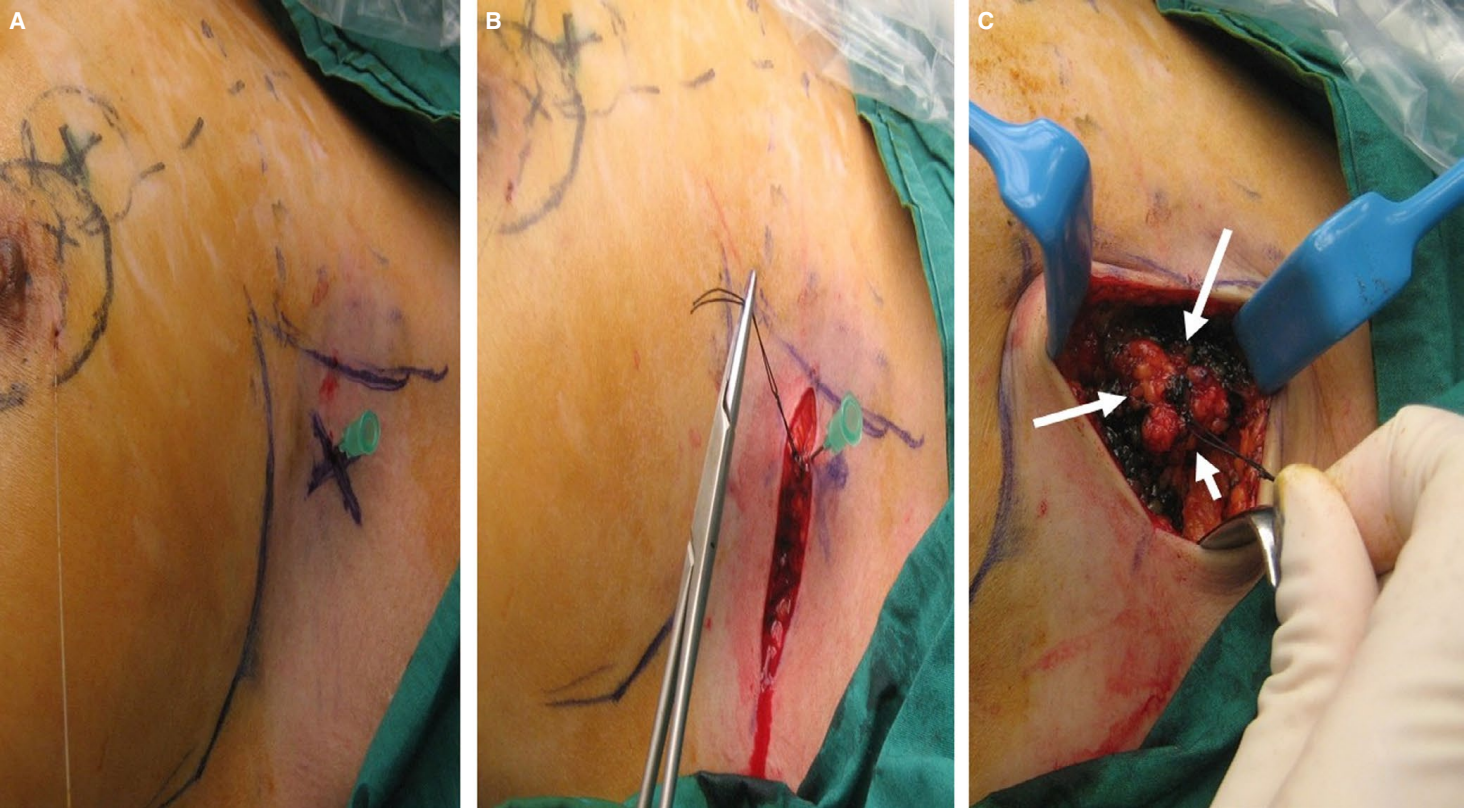
A, The clipped node was localized by placing a 21 G needle perpendicular to skin at the cross marking. B, After raising the skin flaps, the needle was removed after a stitch had been placed at the needle site. C, With the stitch as the center, a 1 cm all‐round margin was marked out with blue ink (arrows) and resection along the blue ink performed

The clip skin‐marked position was checked again after the patient underwent general anesthesia, with the arm abducted at 90 degrees. After cleaning and draping the patient, a 21 G needle was inserted perpendicularly into the skin at the skin‐marked site (Figure 2A).

An axillary crease incision was made and the skin flaps raised, taking care not to displace the 21 G needle. After raising the skin flaps, a stitch was placed at the site of the 21 G needle and the needle subsequently removed (Figure 2B).

Using the stitch as the reference point, a 1 cm all‐around margin was marked out and the axillary tissue was resected based on this marking (Figure 2C). The depth of resection was based on the distance from the clipped node to the skin as measured by ultrasound.

**Figure 3:**
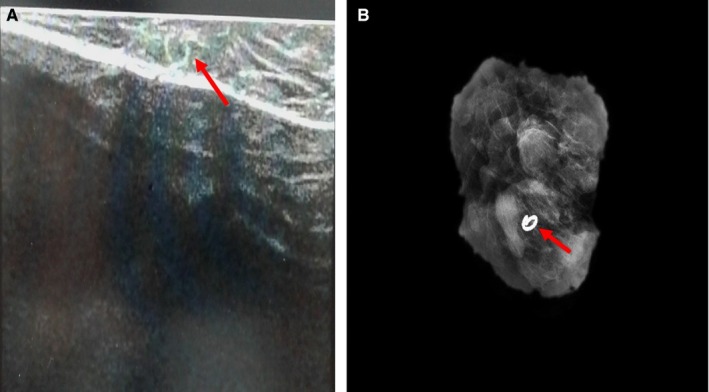
A, Ultrasound image and (B) X ray of the resected clipped node specimen showing the UltraCor Twirl clip (arrow)

The resected clipped node specimen was then checked with ultrasound and X‐ray to confirm the presence of the clip (Figure 3A,B). In addition, histological assessment of the clipped node was performed.

The patient then proceeded with the planned breast surgery and ALND.

### 2.2 Statistical analysis

Patients' demographics and characteristics of the clipped nodes were summarized as frequencies and percentages. In addition, the sonographic features of the skin‐to‐clip distances and lymph node sizes obtained before and after chemotherapy were summarized using their means and medians. Fisher's exact test was used to compare success rates among the various clip types. Because each clip was removed individually and independently of other clips in the same patient, clip removals were viewed as independent events. *P* < .05 was considered as statistically significant. SAS v9.4 (SAS Institute Inc) was used to perform all analyses.

This study obtained SingHealth Centralised Institutional Review approval (CIRB Ref: 2017/2037) and all patients gave written informed consent. This study was registered with ClinicalTrials.gov (identifier: NCT03878017).

## 3 RESULTS

A total of 25 clipped nodes in 14 patients were prospectively assessed. Mean/median age was 55.5/57.5 (range, 27‐74 years). In this study, 78.6% of patients had body mass index [BMI] <25 kg/m^2^ (Table 1). Sonographically, the mean/median breast tumor size was 47.6/40.5 mm (range, 15‐93 mm). 7, 3, and 4 patients had 1, 2, and 3 abnormal lymph nodes seen on ultrasound, respectively; however, percutaneous biopsy revealed 20 of the 25 lymph nodes to be malignant, 3 to be benign, 1 nondiagnostic and 1 with atypical cells; 92.9% and 57.1% of patients had invasive ductal carcinomas and grade III tumors, respectively. 50% of patients had a positive HER2. After NACT, of the 20 clipped metastatic nodes, 45% of clipped nodes achieved ypN0 (Table 2), 71.4% and 28.6% patients underwent mastectomy and breast conservation, respectively.

**Table 1:** Demographics of the patients in the study

Clinical features	No. (%) of patients. N = 14
Age (y)	
≤50	5 (35.7)
>50	9 (64.2)
BMI (kg/m^2^)	
<18.5	2 (14.3)
18.5‐24.9	9 (64.3)
25‐29.9	2 (14.3)
>/=30	1 (7.1)
Sonographic features	
Breast tumor size (mm)^a^	
≤20	2 (14.2)
>20 to ≤50	6 (42.9)
>50	6 (42.9)
No. of abnormal lymph nodes on ultrasound	
1	7 (50)
2	3 (21.4)
3	4 (28.6)
Pathological features	
Breast tumor histology	
Ductal	13 (92.9)
Lobular	1 (7.1)
Grade	
I	0 (0)
II	6 (42.9)
III	8 (57.1)
Estrogen receptor (ER)	
Positive	10^b^ (71.4)
Negative	4 (28.6)
Progesterone receptor (PR)	
Positive	7^b^(50)
Negative	7 (50)
Human epidermal growth factor receptor 2 (HER2)	
Positive	7 (50)
Negative	7 (50)
ypT	
ypTpcr	1 (7.1)
ypTis	3(21.4)
ypT1	5 (35.7)
ypT2	2 (14.3)
ypT3	2 (14.3)
ypT4	1 (7.1)
ypN	
ypN0	5 (35.7)
ypN1	4 (28.6)
ypN2	5 (35.7)
ypN3	0 (0)

a If multifocal/centric disease was present sonographically, the breast size measurement will be based on the largest size of all lesions.

b 2 were weakly positive.

**Table 2:** Characteristics of the 25 clips

Clinical features	No. (%) of clips successfully removed (N = 21)	No. (%) of clips missed (N = 4)	*P* value^a^
Type of clip			.0103
UltraCor Twirl	13 (61.9)	0 (0)	
HydroMARK	7 (33.3)	2 (50)	
UltraClip Dual Trigger	1 (4.8)	1 (25)	
UltraClip	0 (0)	1 (25)	
Sonographic features			
Pre‐NACT clipped node size (mm)^b^			.7870
<10	5 (23.8)	2 (50)	
10‐20	10 (47.6)	1 (25)	
>20	6 (28.6)	1 (25)	
Pre‐ NACT clipped node cortex size (mm)			1.0000
<5	10 (47.6)	1 (25)	
5‐10	7 (33.3)	2 (50)	
>10	4 (19.1)	1 (25)	
Post‐NACT clipped node size (mm)^b^			.7652
<5	9 (42.9)	1 (25)	
5‐9	9 (42.9)	3 (75)	
>9	3 (14.2)	0 (0)	
Post‐NACT clipped node cortex size (mm)			.1799
<0.5	9 (42.9)	0 (0)	
0.5‐1	2 (9.5)	0 (0)	
>1	10 (47.6)	4 (100)	
Skin to clip distance postchemotherapy (cm)			.0167
<1.0	4 (19.1)	0 (0)	
1‐1.5	12 (57.1)	0 (0)	
>1.5	5 (23.8)	4 (100)	
Pathological features			1.0000
ypN^c^			
ypN0	8 (44.4)	1 (50)	
ypN+	10 (55.6)	1 (50)	

Abbreviation: NACT, Neoadjuvant chemotherapy.

a Fisher's exact test.

b Based on the largest size dimension.

c Based on 18 metastatic lymph nodes.

All patients underwent the clip insertion with no complications. 13 UltraCor Twirl, 9 HydroMARK (4 of design 3 and 5 of design 4), 2 UltraClip Dual Triggers and 1 Ultraclip were used (Table 2). The mean/median pre‐NACT clipped node and cortex sizes were 16.88/14 mm (range: 5‐47 mm) and 6.66/5.80 mm (range: 2.1‐20.7 mm), respectively.

Post‐NACT, the mean/median clipped node and cortex sizes were 5.12/5.0 mm (range, 0‐30 mm) and 1.48/1.2 mm (range, 0‐7.6 mm), respectively. In nine clipped nodes, no residual lymph node was visible on ultrasound and 88.9% of these nodes had been clipped with the UltraCor Twirl marker. The mean/median distance from the skin to the clip measured on ultrasound postchemotherapy was 1.66/1.66 cm (range, 1.61‐1.7 cm) and 1.32/1.3 (0.66‐2.2 cm) for the 4 missed clips and the 19 successfully removed clips, respectively. There was no migration of clips noted based on histological correlation.

The UltraCor Twirl marker had the best ultrasound visibility and was well identified on ultrasound in more than half of the twirl marker cases 7/13, whereas hydroMARK, UltraClip Dual Trigger and UltraClip were only moderately or poorly identified. UltraCor Twirl, hydroMARK, UltraClip Dual Trigger, and UltraClip were moderately visualized in 5/13, 2/9, 2/2, and 0/1, respectively. In cases where clips were not confidently identified, previously recorded sonographic distance measurements between the various clips were used as an aid to locate the clips. If post‐NACT magnetic resonance imaging was performed, it was also used as an adjunct to more confidently identify the clips in these cases. With these methods, all clips were eventually identified and locations skin‐marked preoperatively.

Using SMART, removal rates for UltraCor Twirl, hydroMARK, UltraClip Dual Trigger, and UltraClip clipped nodes were 13/13 (100%), 7/9 (77.8%), 1/2 (50.0%), and 0/1 (0%), respectively, with an overall removal rate of 84%. The remaining four missed clips were all retrieved in the ALND specimens. The four missed clips all occurred in patients containing three clipped nodes, for which initial visualization difficulties were experienced for hydroMARK, UltraClip Dual Trigger, and UltraClip, with a mean skin to clipped node distance of 1.66 cm.

Patients' demographics and lymph node sizes pre‐ and postchemotherapy did not reveal any statistically significant association with the success of SMART. However, comparisons among type of clips used (*P* = .0103) and depth of the clips (*P* = .0167) (Table 2) did show statistical significance. The number of clipped nodes per patient showed a significant difference (*P* = .0010) with all patients having three clips experiencing one failure.

There were no postoperative complications.

## 4 DISCUSSION

Our initial experience with the SMART novel technique revealed an overall successful clipped nodes removal rate of 84% with no postoperative complications. The removal rate was 100% with the UltraCor Twirl Marker which had the best ultrasound visibility. Use of other markers, which were not so readily visible, and placement of three clips in a single patient and deeper seated clips were factors associated with failure of clipped node removal. To the best of our knowledge, this is the first study introducing skin marking in the axilla as a form of localization. It is also one of the few studies evaluating the ultrasound visibility of different clips in the axillary lymph nodes post‐NACT.

Accurate identification of the clip remained a crucial factor in determining the precise placement of the localizing agent or skin mark and hence the success of clipped node removal. However, ultrasound visibilities of clips in the axillary lymph nodes post‐NACT have been suboptimal with reported rates of 72%‐83.3%.^11^, ^12^ This is because chemotherapy can cause shrinkage and fibrosis of the lymph nodes resulting in loss of hypoechogenicity of the lymph nodes upon ultrasound, which makes it challenging to identify the tiny clips against a background of echogenic fat.

As a result, other imaging modalities,^12^ such as computed tomography (CT), have been deployed to aid in identifying clips and facilitate clip localization using the wire.^13^ However, a CT carries radiation risk.

All localizing agents have their own limitations and disadvantages. The wire has wire‐related complications such as displacement and breakage and may not be technically feasible in all cases. The radioactive seed carries radiation exposure and requires radiation safety precautions and handling.^14^ Other promising modalities include a radar breast localization system (Savi Scout),^8^ Magseed^9^ or radiofrequency identification technology^15^ predominantly used for breast lesions. These devices have no radiation risk and can avoid the use of wire. However, these agents are costly and not readily available worldwide. In addition, Magseed is not compatible with standard surgical instruments. Charcoal tattooing of positive lymph nodes at the time of biopsy has also been reported.^16^, ^17^ However, there may be a small risk of charcoal migration, potential nonvisualization of the tattoo at the time of surgery.

To overcome some of the limitations associated with the current localization agents, the SMART innovation was conceived. The SMART success rate is largely a consequence of the ultrasound visibility of the clip which has been greatly improved with the use of a newly available biopsy marker, the UltraCor Twirl marker, which was well and moderately identified sonographically in nearly 100% of our cases. The UltraCor Twirl marker is more distinctly ultrasound visible, compared to the traditional markers, because of the marker's unique twirled ring shape when it is deployed.^18^ The result is a distinctive sonographic ring appearance instead of a straight metallic line as witnessed with the other markers.

With improved clip visibility on ultrasound, the clipped nodes could be excised in a similar fashion as nonpalpable clipped breast lesions using ultrasound guidance alone.^7^ In fact, intraoperative ultrasound‐guided excision of axillary clipped node post‐NACT had been reported to be feasible and safe.^19^ In our case however, we advocated a multidisciplinary approach of the radiologists preoperatively placing a skin mark to further delineate the location of the clip more precisely. To ensure maximal visualization of the clips, the clips were placed in the cortex to enhance the visibility of the echogenic clip against the hypoechoeic background of the cortex. The perpendicular distance of the clip to the skin, measured with the linear transducer and with the arm abducted at 90°, was used for future identification of the clip post‐NACT. After NACT, if the original clipped node was no longer visible and the clip was difficult to visualize, review of prior axillary ultrasound images and available chest cross‐sectional imaging studies could assist in locating the clip.

As the skin is mobile over the axilla area, it is important that the skin marking is performed with the patient's arm in the position mimicking the operative condition. After general anesthesia, the skin was then immobilized using the 21 G needle which was inserted perpendicularly at the marked point. When raising the skin flaps, and especially excising the clipped nodes individually in patients with multiple clipped nodes, care needs to be taken not to displace the remaining needles. This might explain why this technique was suboptimal in patients with three clipped nodes in our study.

In addition, our technique would require the prerequisite of the surgeon being trained in ultrasound imaging and to have an ultrasound machine in the operating theatre. The ultrasound could confirm again the location of the clipped node to be at the skin mark. Also, should the clipped node not be found after first the attempt, a repeat localization of the clipped node can be attempted intraoperatively.

The removal rate of SMART in our study was comparable to the rates reported for other localization agents. The radioactive seed, wire and tattooing techniques had a removal rate of nodes of 97%‐100%,^6^, ^20^ 71%‐100%,^11^, ^13^, ^21^, ^22^ and 83%‐100%,^16^, ^17^ respectively.

The type of clip used was a statistically significant factor determining the success of SMART in the removal of clipped lymph nodes, with a significant difference between UltraCor Twirl and other clips (*P* = .0103). This was attributed to the better ultrasound visibility of the UltraCor Twirl marker which could still be identified distinctly in cases without visible lymph node on ultrasound postchemotherapy.

Skin Mark clipped Axillary nodes Removal Technique was also not optimal in patients with three clipped nodes. This could be explained by a possible displacement of the remaining 21 G needles during dissection of the individual clipped lymph node. However, in the current literature, it appears that clipping the most suspicious lymph node may suffice to improve the false negative rate of SLNB post‐NACT,^5^ so the need for multiple clipped nodes may not be required.

In addition, the skin to clip distance was a statistically significant factor, suggesting that SMART may not be so suited for removal of deeply seated clipped nodes. Little literature exists on the skin distance of clipped nodes postneoadjuvant chemotherapy. In an American study of 24 patients,^23^ the sonographic skin to node distance was reported as between 8 and 35 mm in 95.8% of patients. In comparison, the skin to clipped node distance in our Asian study, with BMI of <25 kg/m^2^ in 78.6% of patients, was between 6.6 and 22 mm. As a result, SMART may still be applicable in removal of some clipped nodes, especially the not so deeply seated clipped nodes in the Asian population setting. This could hence reserve the use of the conventional localizing agents to the deeply seated clipped nodes. This finding will however require further validation in larger studies.

Advantages of this technique are the radiation‐free, wireless and noninvasive nature of preoperative localization. It is significantly cheaper than other localizing devices. In centers where these localizing devices are not available, our novel technique offers another way of removing the clipped nodes, allowing the patient a trial of axillary preservation and avoiding the complications associated with ALND.

Limitations of our study included a small sample size and as all patients underwent ALND, we were unable to assess the compatibility of our novel technique with SLNB. However, if SLNB were to be attempted with SMART, we would recommend the removal of clipped nodes first before proceeding with SLNB, to avoid the displacement of the needles. The compatibility of SMART with SLNB will be assessed in the next phase of the study. It is also not optimal in patients with three and/or deeply seated clipped nodes.

The duration of SMART, number of lymph nodes in each clipped node specimen and long‐term complications will be investigated in future studies.

In conclusion, our novel technique (SMART), coupled with the use of UltraCor Twirl marker, in patients with fewer than three clipped nodes which were not deeply seated, could allow 100% removal of the clipped nodes safely and avoid the cost and complications associated with current localization modalities.

## CONFLICT OF INTEREST

The authors declare no conflict of interest.
